# Vertebral fractures and self-perceived health in elderly women and men in a population-based cross-sectional study: the Tromsø Study 2007–08

**DOI:** 10.1186/1471-2318-13-102

**Published:** 2013-09-30

**Authors:** Svanhild Waterloo, Anne J Søgaard, Luai A Ahmed, Elin Damsgård, Bente Morseth, Nina Emaus

**Affiliations:** 1 Department of Community Medicine, Faculty of Health Sciences, University of Tromsø, 9037, Tromsø, Norway; 2 Norwegian Institute of Public Health, Oslo, Norway; 3 Department of Health and Care Sciences, University of Tromsø, Tromsø, Norway; 4 Regional Centre for Sport, Exercise, and Health – North, Faculty of Health Sciences, University of Tromsø, Tromsø, Norway

**Keywords:** Vertebral fractures, Health-related quality of life (HRQL), Self-perceived health, Pain, Population-based study

## Abstract

**Background:**

Health-related quality of life (HRQL) may be associated with increased mortality in the elderly. The effect of prevalent vertebral fractures on HRQL in elderly women and men is not well described. The purpose of this study was to examine the association between prevalent vertebral fractures and back pain, neck pain, and HRQL in elderly women and men, and to study possible gender differences in the reported pain and HRQL.

**Methods:**

Information on prevalent vertebral fractures was ascertained by a vertebral fracture assessment (VFA) method (dual-energy X-ray absorptiometry (DXA), GE Lunar Prodigy) in 2887 women and men, mean age 65.4 (SD 9.4) who participated in the population-based Tromsø Study which was conducted in 2007–08. Bone mineral density (BMD; g/cm^2^) was measured by DXA at the femoral sites. Self-reported HRQL was assessed using the standardized measures EQ-5D-3 L and EQ VAS from the EuroQol Group. Lifestyle information was collected by questionnaires. The association between vertebral fractures and pain was analyzed using logistic regression, between vertebral fractures and EQ-5D-3 L and EQ VAS scores by multiple regression analyses.

**Results:**

In women, presence of vertebral fractures was associated with an increased risk of back pain with an OR of 1.76 (95% CI: 1.24 – 2.50) after adjustments for age, height, weight, and BMD. Women with vertebral fractures had lower EQ-5D-3 L scores (p < 0.001) than women without vertebral fractures, also after adjustments. These associations were not present in men. Type of fracture was not associated with EQ-5D-3 L scores, but increasing numbers (p < 0.001) and severity of fractures (p < 0.002) were associated with decreasing EQ-5D-3 L score in women.

**Conclusion:**

Prevalent vertebral fractures are associated with increased risk of back pain and reduced HRQL in postmenopausal women, but not in men.

## Background

Health-related quality of life (HRQL) may be associated with increased mortality in the elderly [1,2]. Osteoporotic fractures, which are prevalent in the elderly [3] are also associated with increased morbidity and mortality [3,4]. Vertebral fractures are one of the most frequent and serious osteoporotic fractures [5]. According to the literature, as much as one third of all vertebral fractures are never clinically diagnosed [6-10], often because of methodological problems. Vertebral fractures may also be asymptomatic [11], but it has been well documented in several studies that osteoporotic vertebral fractures may be associated with pain [12-14]. There is an abundance of literature indicating an association between vertebral fractures and impaired quality of life in health matters [10,15-20]. Whether these associations are connected to the number of fractures, type of fractures, or severity of fractures is less explored. Elderly people may explain their back pain as simple wear and tear, without considering vertebral fractures as a possibility. Most of the recent studies on vertebral fractures and HRQL include small patient populations [10,14] or postmenopausal women only [10,12,14,16,20,21]. These studies indicate that in women, HRQL and daily life are strongly affected by the impact of the vertebral fracture.

To our knowledge, the impact of vertebral fractures on HRQL comparing the two sexes has not been studied. In a general population of elderly men and women, we therefore wanted to:

1) Examine the association between prevalent vertebral fractures and neck and back pain and measures of HRQL.

2) Examine if there are any gender differences in the association between vertebral fractures, reported pain and HRQL.

## Methods

### Study population

The present study is part of the Tromsø Study; design and protocol has been described in detail elsewhere [22]. In brief, the Tromsø study is a longitudinal, population-based survey in Tromsø, a city in northern Norway with approximately 70000 inhabitants. The Tromsø Study has conducted six repeated surveys from 1974 (Tromsø I) through 2007–08 (Tromsø VI) [23]. Participation rates have ranged from 77% in Tromsø I-V to 66% in Tromsø VI [23]. Each survey consists of two phases, with the most basic examinations performed in phase 1 and after one month more extensive examinations performed in phase 2 in a random sub-sample of the cohort. The present study utilizes data from Tromsø VI in 2007–08, where height and weight measurements and questionnaire data are derived from phase 1 and vertebral morphometry and bone mineral density (BMD) measurements are from phase 2 of the study.

A total of 6054 men (attendance rate 62.9%) and 6930 women (attendance rate 68.4%) attended phase 1 of the 2007/08 survey. Among those, a total of 11484 subjects were invited for phase 2, and 3141 men and 4166 women attended, providing an attendance rate of 63.6% in phase 2. Among the phase 2 attendants, all persons who had valid BMD measurements from the 2001/02 survey were invited for BMD measurements of the hip, i.e., a dual femur scan, and altogether 3854 persons attended this examination. Among these 3854 persons, a lateral vertebral assessment (LVA), also called a vertebral fracture assessment (VFA) was performed in a randomly selected group of 2894 persons. Seven blurred VFA scans had to be excluded, leaving 2887 persons, 1206 men and 1681 women, with clearly measurable VFA scans, providing information utilized in the present study.

The Regional Committee of Medical Research Ethics and the Norwegian Data Inspectorate recommended the study, and each participant gave written informed consent prior to inclusion.

### Ascertainment of vertebral fracture

The VFA was conducted using the GE Lunar Prodigy (Lunar Corporation, Madison, WI, USA, version 12.20) morphometric software. Vertebral morphometry is a quantitative method developed for identification of osteoporotic vertebral fractures based on the measurement of vertebral heights, identifying the anterior, middle, and posterior heights of each vertebra. Depending on their relative relations according to a given reference, the software identifies three types of fractures: wedge, biconcave, and compression. The fractures are classified according to three degrees of severity, ranging from mild through moderate to severe [8,24]. Some authors suggest that the spinal radiograph is the gold standard for the diagnosis of vertebral fractures [9,25], while others would argue that there is no agreed-upon gold standard for the definition of vertebral fractures [26-28]. The morphometric method is generally recognized as being easy, precise and using low radiation exposure [29-31], with high precision in measuring moderate and severe deformities [9]. Here, three technicians did the scanning according to a standardized protocol, and one of them (the first author) performed the quality assessment of the total material afterwards. For precise analysis of the VFA, a random sample of 50 participants was re-analyzed. The mean intra-class correlation coefficient was 0.84 for average height of the vertebrae [22]. In this study, we use information concerning presence or no presence, type, number, and severity of vertebral fractures.

### Bone mineral density

BMD is a strong predictor of fracture risk [32,33]. BMD expressed as g/cm^2^ was measured at the total hip and femoral neck by dual-energy X-ray absorptiometry (DXA), using the same densitometry as for the VFA (GE Lunar Prodigy). Daily phantom measurements were performed throughout the survey and no densitometer drift was detected. The short term in vivo precision error was 1.2% and 1.7% for total hip and femoral neck measurements, respectively [34]. For the main analyses of this study, we have included BMD measurement of the total hip, where 2791 valid measurements were available at either the left or the right hip.

### Questionnaire

Data on lifestyle variables were collected through questionnaires in both phases of the study, including information on socio-demographics as well as smoking habits, leisure time physical activity, education, neck and back pain, and HRQL. Smoking status was classified into two categories: former and never smokers as “not smoking” and smokers as “smoking”. The question on physical activity level during the last year had four alternatives from sedentary through moderate and active to highly active. The sedentary and moderate were categorized into a “low active group” and the active and highly active into a “high active” group. Education was categorized into three levels: 1) primary school only (i.e. 7 years), 2) up to 4 years more than primary school, and 3) more than 4 years after primary school. Neck pain and back pain were recorded as “yes” or “no” based on three alternatives: “no complaints” (no), “some complaints” or “severe complaints” (yes).

### Assessment of health-related quality of life

HRQL is the main outcome in the present study and was measured by EQ-5D-3 L and the EQ VAS scores. The EQ-5D-3 L is a standardized measure of health status developed by the EuroQol Group [35]. The HRQL assessment provides a simple descriptive profile (EQ-5D-3 L) and a single index value for health status (EQ VAS) that is suited for use in population health surveys [10,36,37]. EQ-5D-3 L consists of five questions on the following dimensions (5D): mobility, self-care, usual activities, pain/discomfort, and anxiety/depression, each scored on three levels (3 L): no problems, some problems, extreme problems. The EQ VAS shows the respondent’s self-rated health on a vertical, visual, thermometer-like analogue scale where the endpoints are labeled “Best imaginable health state” at the top (100) and “Worst imaginable health state” (0) at the bottom. In a systematic literature review of self-assessed health instruments, the EQ-5D was one of the recommended generic health instruments for use in older people [38]. As our population had a mean age of approximately 65 years (those without a fracture) and approximately 70 years (those with a fracture), we found the EQ-5D useful for our purposes. We have applied the UK time-trade-off (TTO) tariff, the scoring algorithm most often used.

### Data analysis

Each individual was classified as having a vertebral fracture if there was the presence of at least one fracture as described in the “Ascertainment of vertebral fracture” section. Relevant variables were compared between those with and without prevalent vertebral fractures in univariate analysis (using chi-square testing for categorical variables and independent t-tests for continuous variables), for women and men, separately. The association between vertebral fractures and pain (neck pain, back pain) or quality of life (EQ-5D-3 L, EQ VAS) was examined using logistic or linear regression analyses, respectively, adjusting first for age, thereafter for age, height, weight and BMD. For assessment of the effect of fracture severity, we examined if EQ-5D-3 L and EQ VAS differed between types of fractures (wedge, biconcave, and compression) and numbers of fractures (categorized into 0, 1, 2, and 3 or more) using ANOVA, applying the Bonferroni for multiple correction test. For further assessment of severity, participants were categorized into three groups: no or mild fractures, moderate, and severe fractures, without consideration of type of fracture, and ANCOVA was applied to examine if EQ-5D-3 L and EQ VAS differed between these groups. All statistical analyses were performed with the IBM SPSS statistical package version 19. A *p-*value below 0.05 was considered significant.

## Results

The study population included a total of 2887 individuals (1206 men and 1681 women) aged between 38 and 87 years. We excluded all participants below the age of 50 years from the analyses and remained with 1177 men and 1615 women in analyses. Overall, approximately 64% of men and 61% of women reported that they were in good health, but a majority (more than 78%) was in the low physical activity group. In participants 50 years and older, vertebral fractures were found in 165 (14%) men and 197 (12.2%) women (p = 0.09). Baseline characteristics of participants stratified by fracture status and sex are shown in Table 1. Men and women with vertebral fractures were older and had significantly lower total hip BMD compared with participants without fracture. In addition, women with fractures were shorter and weighed less compared with women without.

**Table 1:** Descriptive statistics by gender and morphometric vertebral fracture, the Tromsø Study 2007-08

**Gender and factor**	**No fracture**	**Vertebral fracture**	**P-value**
**Men (N)**	**1012**	**165**	
Age (years); mean (SD)	65.4 (8.8)	69.1 (9.1)	<0.001
Weight (kg); mean (SD)	84.3 (12.2)	82.7 (11.5)	0.116
Height (cm); mean (SD)	175.3 (6.5)	174.4 (6.8)	0.096
BMI (kg/m^2^); mean (SD)	27.4 (3.5)	27.2 (3.4)	0.444
Total hip BMD (g/m^2^); mean (SD)	1.030 (0.14)	0.977 (0.16)	<0.001
Smoking status			0.486
Daily smoking; n (%)	152 (15.9%)	25 (15.6%)	
No smoking; n (%)	847 (84.1%)	135 (84.4%)	
**Women (N)**	**1418**	**197**	
Age (years); mean (SD)	65.7 (8.4)	70.8 (8.2)	<0.001
Weight (kg); mean (SD)	71.1 (12.4)	68.4 (12.7)	0.005
Height (cm); mean (SD)	162.4 (6.3)	160.3 (7.1)	<0.001
BMI (kg/m^2^); mean (SD)	27.0 (4.6)	26.6 (4.5)	0.298
Total hip BMD (g/m^2^); mean (SD)	0.906 (0.13)	0.829 (0.11)	<0.001
Smoking status			0.370
Daily smoking; n (%)	252 (18%)	37 (19.3%)	
No smoking; n (%)	1138 (82%)	154 (80.7%)	

1) In analyses: 1615 women and 1177 men above 50 years of age.

2) Comparison of the variables between participants with and without vertebral fractures in univariate analyses, using chi-square testing for categorical variables and independent t-tests for continuous variables.

### Fracture vs. no fracture

As displayed in Table 2, men with vertebral fractures did not report more back or neck pain compared with men without fractures, and EQ-5D-3 L and EQ VAS scores did not differ between the two groups. Adjusting for age alone, or age, height, weight, and total hip BMD did not change the results (Table 3). Women with vertebral fractures reported significantly more back pain (p = 0.003) and had lower EQ-5D-3 L scores (p < 0.001) and EQ VAS scores (p = 0.014) than women without vertebral fractures (Table 2). Back pain was also significantly different in women with and without fractures adjusting for age alone (p = 0.004) and for age, height, weight, and total hip BMD (p = 0.001) with an OR of 1.76 (95% CI 1.24 – 2.50) in women with vertebral fractures (Table 3). Also, EQ-5D-3 L was significantly lower in women with vertebral fractures, whether adjusting for age alone or adjusting for age, height, weight, and total hip BMD (p < 0.001) (Table 3). In univariate analyses, EQ VAS was lower in women with fractures (p = 0.014) (Table 2). Adjusting for age, EQ VAS score was no longer significantly different between women with and without fractures (p = 0.058). Further adjustments including height, weight, and total hip BMD confirmed this result (p = 0.058) (Table 3).

**Table 2:** The association between prevalent vertebral fractures and back pain, neck pain, and health-related quality of life (HRQL) in univariate analyses, the Tromsø study 2007-08

**Gender and factor**	**No fracture**	**Vertebral fracture**	**P-value**
**Men (N)**	**1012**	**165**	
Back pain; n (%)	145 (85%)	25 (15%)	0.429
Neck pain; n (%)	124 (89%)	16 (11%)	0.211
EQ-5D-3 L; mean (SD)	0.86 (0.17)	0.86 (0.16)	0.988
	(n = 931)	(n = 145)	
EQ VAS; mean (SD)	77.1 (15.1)	76.7 (12.5)	0.845
	(n = 426)	(n = 61)	
**Women (N)**	**1418**	**197**	
Back pain; n (%)	321 (84%)	63 (16%)	0.003
Neck pain; n (%)	313 (87%)	47 (13%)	0.315
EQ-5D-3 L; mean (SD)	0.82 (0.18)	0.74 (0.25)	<0.001
	(n = 1254)	(n = 164)	
EQ VAS; mean (SD)	75.7 (17.4)	70.0 (17.9)	0.014
	(n = 559)	(n = 65)	

1) The specific number of participants with HRQL- information is given in brackets.

2) Comparison of the variables between participants with and without vertebral fractures in univariate analyses, using chi-square testing for categorical variables and independent t-tests for continuous variables.

**Table 3:** The association between prevalent vertebral fractures and back pain, neck pain and health-related quality of life (HRQL) in multivariate analyses, the Tromsø study 2007-08

**Men**	**OR (95% CI)**	**P-value**
Vertebral fractures and back pain	1.09 (0.67 – 1.75)	0.735
Vertebral fractures and neck pain	0.82 (0.47 – 1.43)	0.483
**Men**	**Beta**	**P-value**
Vertebral fractures and EQ-5D-3 L	0.008	0.802
Vertebral fractures and EQ VAS	0.013	0.682
**Women**	**OR (95% CI)**	**P-value**
Vertebral fractures and back pain	1.76 (1.24 – 2.50)	0.001
Vertebral fractures and neck pain	1.18 (0.81 – 1.73)	0.381
**Women**	**Beta**	**P-value**
Vertebral fractures and EQ-5D-3 L	−0.107	<0.001
Vertebral fractures and EQ VAS	−0.077	0.058

1) The presented results after adjustments for age, height, weight and total hip BMD.

2) In women, presence of vertebral fractures was associated with an increased risk of back pain with an OR of 1.76 (95% CI: 1.24 – 2.50).

3) In women, presence of vertebral fractures was associated with a decreasing EQ-5D-3 L score.

### Type of fracture

There was no association between type of fracture and pain experience in men (p > 0.348) nor in women (p < 0.122). In men, EQ-5D-3 L differed between the groups (p = 0.024) so that the scores were 0.84, 0.91, and 0.83 in those with wedge, biconcave, and compression fractures, respectively. However, after adjusting for age (p = 0.153) and adjusting for age, height, weight, and total hip BMD (p = 0.258), the results were no longer significant. With EQ-5D-3 L scores of 0.74, 0.71, and 0.79 in those with wedge, biconcave, and compression fractures, respectively, there was no association between EQ-5D-3 L and types of fracture in women (p = 0.322). EQ VAS was not significantly different between types of fractures (p > 0.42) in either sex. Adjustments for age, height, weight, and BMD did not change the results.

### Number of fractures

There was no association between number of fractures and neck pain in either sex (p > 0.53) and number of fractures and back pain in men (p = 0.44). In women, the percentage reporting back pain increased with increasing number of fractures (p = 0.015). With EQ-5D-3 L scores of 0.87, 0.87, 0.84, and 0.89 in those with 0, 1, 2, and 3 and more fractures, respectively, there was no association between number of fractures and EQ-5D-3 L in men (p = 0.740). In women, EQ-5D-3 L differed between the groups (p < 0.001), with EQ-5D-3 L scores of 0.82, 0.74, 0.74, and 0.71, in those with 0, 1, 2, and 3 or more fractures, respectively. Adjusting for age alone, or for age, height, weight, and total hip BMD, did not change the results (p < 0.001). There was no association between number of fractures and EQ VAS in men (p = 0.239) or in women (p = 0.072). Adjusting for age did not change this result, nor did further adjustments.

### Severity of fractures

**Figure 1:**
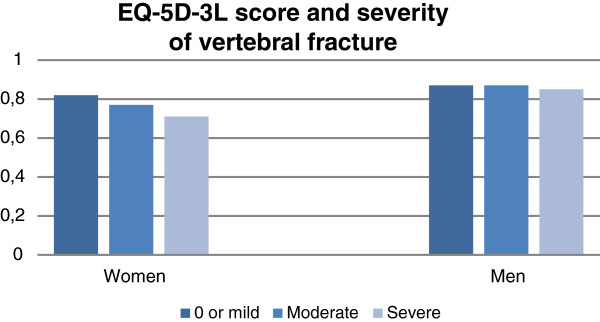
EQ-5D-3 L score was associated with severity of vertebral fracture in women but not in men, the Tromsø Study 2007–08.

There was no association between severity of fractures and neck pain in either sex (p > 0.12). There was no association between severity of fractures and back pain (p = 0.541) and severity of fracture was not associated with EQ-5D-3 L (p = 0.711) or with EQ VAS (p = 0.422) in men. In women, severity of fractures was associated with back pain (p = 0.015) and the mean EQ-5D-3 L score was 0.82, 0.77, and 0.71 in those with 0 or mild, moderate, and severe fractures, respectively (p < 0.001) (Figure 1). Adjusting for age alone did not change this association (p < 0.001), nor did further adjustments for age, height, weight, and total hip BMD (p = 0.002). In women, EQ VAS also differed between severity types (p = 0.018). The difference was still statistically significant when adjusting for age, height, weight, and total hip BMD (p = 0.044).

## Discussion

In this population-based study, we found a prevalence of vertebral fracture which was quite similar in men and women above the age of 50 years, but women with prevalent vertebral fractures reported more often back pain and had poorer EQ-5D-3 L and EQ VAS scores than women without vertebral fractures. In men, no such associations were observed. Type of fracture (wedge, biconcave, or compression) did not influence the results, but number of fractures (1, 2, 3 or more) and severity of fractures (mild, moderate, or severe) influenced the scores in women.

One explanation of the gender difference in results could be that men tolerate a higher degree of physical pain than women do. Pain assessment has always been a challenge and remains so. As pain is both a personal and a subjective matter, researchers have to rely on people’s self-reported experience without any further verification. The nature of pain makes objective measurement impossible [39]. It has for a long time been acknowledged that there are gender differences in pain experiences [40], especially in conditions like chronic back pain [41]. In a review from 2009, Fillingim et al. state that women are at substantially greater risk of many clinical pain conditions, including general musculoskeletal pain, back pain, osteoarthritis, and fibromyalgia [42]. Although we have not been able to find other studies comparing differences in pain sensations among persons with and without vertebral fractures, the findings reported by Fillingim et al. may apply to vertebral fractures as well. Another possible explanation of our findings could of course be that men have more asymptomatic vertebral fractures from earlier ages, as noted by others [31]. Any fracture that occurs in younger years will heal faster and with fewer problems than fractures due to osteoporosis in later stages of life. Because of the study design, this is only a hypothesis, and we will need both follow-up data and studies on younger populations to draw conclusions on this matter.

Decreased health-related quality of life has been reported among postmenopausal women with vertebral fractures [21,43]. In a study by Cockerill et al., recent vertebral fractures were associated with impairment in quality of life, mainly among those who had sustained a previous vertebral deformity (comparable to those with multiple fractures in our study) [15]. It is a great limitation to our study that we do not know *when* the vertebral fractures occurred, as the greatest impact on quality of life most probably is during the first year after fracture, with possible improvements in the second year [10]. As observed by others [44], vertebral fractures that come to clinical attention (“clinical vertebral fractures”) have a significant effect on quality of life. In our cohort, we could not distinguish between clinical and asymptomatic fractures. Interestingly, in a study by Adachi, postmenopausal women with prevalent vertebral fractures had very similar EQ-5D-3 L scores to the scores in the present study, depending on number of fractures [45]. All in all, our findings in women of a clear negative association between vertebral fractures, number of fractures, and severity of fractures and reported back pain and EQ-5D-3 L highlights the effect of vertebral fractures on HRQL in women above the age of 50, which should be studied further.

One of the strengths of the present study is its population based design, a relatively large sample size, and the inclusion of both women and men in the analyses. The cross-sectional design is a huge limitation. We do not know when the observed vertebral fractures occurred, and therefore we do not know the effect of time since fracture on the associations we observed. Follow-up studies are therefore warranted both concerning incidence, the effect of incident vertebral fractures on HRQL, and also on morbidity and mortality in elderly women and men.

## Conclusions

We found, as reported by others, that prevalent vertebral fractures are associated with reduced health-related quality of life in postmenopausal women. The surprising finding was that these associations were not present in men. This may reflect a higher number of asymptomatic fractures in men, or gender differences in pain sensations and pain experience that should be further elucidated.

## Competing interests

The authors declare that they have no competing interests.

## Authors’ contributions

Contributions of the authors to the manuscript included: *Study concept and design*: NE, SW, AJS; *Acquisition of data:* NE, SW, LAA; *Analyses and interpretation of data*: SW, NE, LAA, AJS; *Statistical analyses*: SW, NE; *Critical revision of the manuscript*: SW, LAA, AJS, BM, NE. All authors read and approved the final manuscript.

## Pre-publication history

The pre-publication history for this paper can be accessed here:

http://www.biomedcentral.com/1471-2318/13/102/prepub
